# Characteristics of biochar pellets from corn straw under different pyrolysis temperatures

**DOI:** 10.1098/rsos.172346

**Published:** 2018-08-29

**Authors:** Xianjun Xing, Fangyu Fan, Wen Jiang

**Affiliations:** 1 Advanced Energy Technology and Equipment Research Institute, Hefei University of Technology, Hefei, Anhui 230009, People's Republic of China; 2 School of Chemistry and Chemical Engineering, Hefei University of Technology, Hefei, Anhui 230009, People's Republic of China; 3 National Urban Energy Measurement Center (Anhui), Hefei, Anhui 230009, People's Republic of China; 4 College of Light Industry and Food Science, Southwest Forestry University, Kunming, Yunnan 650224, People's Republic of China

**Keywords:** corn straw, biochar pellets, pyrolysis temperature, characteristics

## Abstract

Biomass resources have the potential to produce clean-energy. However, their physico-chemical properties are inferior to those of coal, and thus, biomass resources are not regarded as ideal feedstock for industrial application. In the present study, the pyrolysis of corn (maize) straw pellets was performed under different temperatures (400, 450, 500, 550 and 600°C) at a 10°C min^−1^ heating rate and 30 min residence time, and the characteristics of biochar pellets were carefully investigated, particularly their elemental composition, hydrophobicity and mechanical resistance. Fourier transform infrared, proximate analysis and scanning electron microscopy were performed. Results indicated that the mass and energy yields of the biochar pellets decreased from 35.46 to 28.65% and from 50.17 to 45.52%, respectively, at increased temperature. Meanwhile, the higher heating value of the biochar pellets increased from 15.45 MJ kg^−1^ in the raw materials to 21.86 and 24.55 MJ kg^−1^ in the biochar produced at 400 and 600°C, respectively. In addition, biochar pellets showed good hydrophobicity, which benefited their storage and transportation, though mechanical resistance decreased. The pellets had compact structures, regular shapes and weakened or no functional groups in contrast with raw pellets, and these properties played important roles in the improvements.

## 1. Introduction

Owing to the increasing demand for fossil fuels and growing environmental pollution levels, biomass resources have been widely used as major sources of renewable energy in many countries [1,2]. However, biomass resources have high moisture content and low bulk energy densities, and exhibit propensity to decay during storage, and thus, their use is limited. Presently, several methods, such as pelletization and pyrolysis [2–4], are used for overcoming these disadvantages and other related problems.

Biomass pelletization is a process in which biomass residues or wastes are compacted into uniformly sized solid fuels either with all kinds of binders or without binders through the application of mechanical force [1,5]. Biomass pellets have low moisture contents, high calorific values, and uniform shapes and demonstrate clean burning. They reduce costs and problems related to transport and storage because of their stable quality, strong weatherability and suitability to long-term storage [6,7]. For the above-mentioned reasons, biomass pellets are recommended in the energy industry. However, the popularization and application of pellets are extremely expensive. In China, corn straw pellets cost about $75 per ton. Therefore, the utilization of pellets must be maximized such that their values are enhanced. In this paper, biochar pellets were produced by pyrolysis technology, and improve the value of biochar pellets and their application compared to raw pellets.

Pyrolysis technology is currently one of the most interesting processes for biomass thermochemical conversion, as it is a sustainable and Earth-friendly approach for energy produce. Biomass decomposes at a temperature range of 400–700°C, in the absence of oxygen during pyrolysis process [8,9]. Meanwhile, biomasses can be transformed into biochar, bio-oil and gaseous products. Gaseous products can be used as fuels because they contain a large number of H_2_ and CO molecules [10]. Likewise, bio-oil can be used as fuels and ingredients for chemical products, such as phenols, organic acids and oxygenates [11]. Biochar can be used as solid fuels and sources of soil amendment according to pyrolysis conditions [8,12]. Pyrolysis technologies are usually divided into flash, fast and slow pyrolysis. Fast and slow pyrolysis have been widely applied in industry. Fast heating rates (10–600°C s^−1^) are conducive to the quick fragmentation of biomass and increases the amount of generated bio-oil and gases, producing less char. In contrast with fast pyrolysis, slow pyrolysis (5–10°C min^−1^), where hot vapour has a longer residence time, produces larger portions of biochar and non-condensable gas.

It was estimated that the volume of the pyrolysis system can be reduced by about 30 times because of the change of bulk density. Experiment results showed that corn straw pellets bulk density is about 600 kg m^−3^; nevertheless, the powder density is about 20 kg m^−3^ via ourselves. Furthermore, biochar pellets have considerably large density, desirable hardness and good hydrophobicity. Using biochar pellets reduces transport and investment costs for fuel storage and process feeding to greater extent than using biochar powder [13–15]. Therefore, the pyrolysation of biomass pellets has obvious advantages, such as decreased equipment cost, simple equipment system and high production efficiency

In the last decade, some studies on thermal behaviour, biochar properties, reaction mechanisms and kinetics of biomass pellets pyrolysis were conducted. Yan *et al*. [16] studied the fuel properties of biochar pellets from Chinese fir sawdust pellets under different temperatures (400, 450, 500, 550 and 600°C), heating rates (2, 6 and 10°C min^−1^) and residence times (60, 120 and 180 min). They found that biochar pellets have high calorific values and improved compression and shatter resistance at 550°C. In addition, the heating rate of 2°C min^−1^ and residence time of 120 min were chosen for better properties of pellets. Zhou *et al*. [17] investigated the pyrolysis behaviour of pelletized municipal solid waste and found that the char yield dropped significantly with increasing temperatures from 450 to 900°C, due to the high content of plastic groups at high decomposition temperatures. Basu *et al*. [18] analysed the effect of torrefaction on density and volume changes of coarse biomass particles. Their results showed that the shrinkage in the radial direction was 3–4%, reduction in longitudinal direction was 6.5–8.8% and mass yield decreased with torrefaction severity. Paulauskas *et al*. [19] observed the same phenomenon as the swelling phenomenon intensified with temperature increase up to 550°C. They also observed the maximum expansion of wood pellets at 250°C. The diameters of the wood pellets increased by 12% of the initial diameter. At temperatures higher than 550°C, the rate of expansion of the wood pellets gradually decreased. Chen & Lin [15] discussed characteristics of pyrolysis products from oil palm fibre pellets in nitrogen and carbon dioxide atmospheres. They found that pellets can be used as feedstock and CO_2_ can be used as a carrier gas for pyrolysis, with the advantage of reducing reactor volume and achieving CO_2_ utilization. Ghiasi *et al*. [20] discussed the merits and defects of torrefaction after densification and those of densification after torrefaction. The results demonstrated that densifying and subsequently torrefying wood chips is energy efficient.

A total of 250 million tons of corn straw are produced annually in China [21]. Currently, corn straw is frequently disposed by burning, which considerably increases the level of environmental pollution. Compared with biochar generated by burning, biochar of corn straw produced by thermochemical means are promising because of their high lignocellulose content [22]. However, to the best of our knowledge, the characteristics of biochar pellets produced by the pyrolysation of corn straw pellets have not been investigated.

For these reasons, we used corn straw pellets as raw materials for biochar pellets in the present study. The purpose of this research was to elevate the fuel properties (mass yield, energy yield, higher heating value (HHV), hydrophobicity and mechanical resistance) of biochar pellets obtained from the pyrolysis technology of corn straw pellets and explore the theoretical basis for future industrial uses of pyrolysis technology from raw pellets.

## 2. Material and methods

### 2.1. Materials

Pellets from corn (maize) straw were purchased at a local factory. The proximate and ultimate analysis results are shown in table 1.

**Table 1.** Basic properties of corn straw pellets and their biochars. Note: Oxygen content is obtained by difference. Deviations are within 5%.

samples	C (%)	H (%)	N (%)	S (%)	O (%)	moisture (%)	volatiles (%)	ash (%)	fixed carbon (%)	HHV (MJ kg^−1^)	mass yield (%)	Energy yield (%)
CSP	40.02	6.01	0.88	0.23	52.86	8.79	65.94	9.14	16.13	15.45	—	—
CSP400	56.46	2.86	1.29	0.18	39.21	4.67	26.95	22.34	46.04	21.86	35.46	50.17
CSP450	57.14	2.54	1.20	0.17	38.95	4.84	18.33	24.39	52.44	23.76	32.23	49.57
CSP500	58.85	2.81	1.17	0.15	37.02	4.53	14.08	25.51	55.88	24.15	31.00	48.46
CSP550	59.29	2.50	1.13	0.17	36.91	4.42	10.66	26.62	58.30	24.49	29.68	47.05
CSP600	60.84	2.15	1.00	0.14	35.87	4.46	7.79	27.56	60.19	24.55	28.65	45.52

### 2.2. Biochar preparation

**Figure 1.**
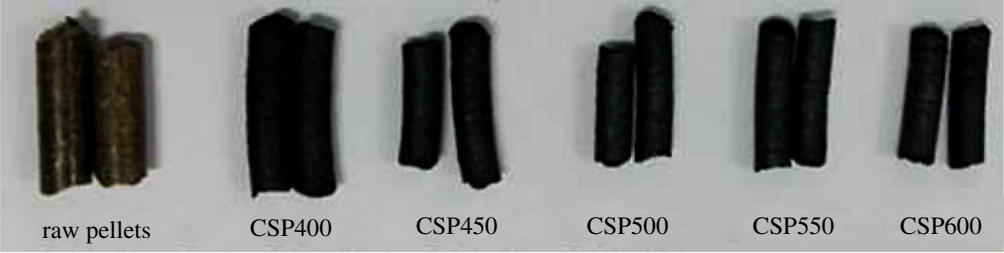
Images of biochar pellets prepared in the present study.

Pyrolysis was conducted at 400, 450, 500, 550 and 600°C at a heating rate 10°C min^−1^ for 30 min in a fixed-bed pyrolysis system (Hefei Ke Jing Materials Technology Co., Ltd, China) under a pure nitrogen atmosphere. The carrier gas that provides the pyrolysis environment was controlled at a flow rate of 100 ml min^−1^. In each experiment, approximately 100.0 g of corn straw pellets were arranged in an alumina boat placed in a quartz tube. To ensure that oxygen was completely removed, we filled the quartz tube with nitrogen at a flow rate of 100 ml min^−1^ for 20 min before the experiment. After cooling down to room temperature, the biochar in the alumina boat was weighed and stored in a desiccator for analysis. Biochar pellets were designated as ‘CSPxxx’, where ‘xxx’ showed the pyrolysis temperature. Figure 1 shows the images of several typical biochar simples prepared in the present study. To ensure the consistency of the experiment and prevent experimental errors caused by varying conditions, we arranged pellets in the same distribution and selected the same location samples in the quartz tube during analysis. Each experiment was conducted in three replicates, and the average values were reported.

### 2.3. Research methodology

For simulation of the actual utilization process, the samples were exposed to air for 24 h before the element analysis and proximate analysis. The elemental contents (C, H, N and S) of the corn straw pellets and biochars were analysed with a Vario Micro Cube elemental analyser (Elementar, Germany). The HHVs of the samples were measured according to the EN 14918 testing standard (combustion under pure oxygen atmosphere at 25°C; an oxygen bomb calorimeter was used; Huadian Analysis Instrument Co., Ltd, China) calibrated with benzoic acid. The mass and energy yield were calculated by equations (2.1) and (2.2) as follows:

2.1 $$\text{Mass}\;\text{yield }(\text{\%})=\frac{m_{\mathrm{biochar}}}{m_{\mathrm{CSP}}}\times 100$$

and

2.2 $$\text{Energy}\;\text{yield}\;(\text{\%})=\frac{\text{HH}{\text{V}}_{\mathrm{biochar}}}{\text{HH}{\text{V}}_{\mathrm{CSP}}}\times \text{mass}\;\text{yield}.$$

The hydrophobicity was expressed by equilibrium moisture content (EMC) and water resistance [23]. The EMC was measured by placing 20.0 g of each sample in two separate large bottles containing saturated sodium chloride and potassium carbonate at experimental temperature of 30°C for 48 h. The relative humidity (RH) of the bottles is approximately 75%, whereas that of the other bottle was 42%. After exposure, the samples were dried in an oven at 105°C for 24 h, the change in the weight before and after drying was calculated as the EMC. Water resistance was analysed. First, dried pellets and biochar pellets were immersed in water for 2 h. Then, the samples were drained by placing them on an adsorbent paper before they were gently placed on an aluminium sheet and exposed to a controlled environment (RH: 48–52% at 22–23°C) for 2 h. Last, the final material was weighed. Change in weight was expressed as the difference between the moisture contents of the final materials and raw samples.

Mechanical resistances were expressed by drop resistance, compression strength and durability. Samples were dropped from 1.85 m height on the concrete floor four times. After being dropped, the samples were screened by using a 2 mm sieve, and particles larger than 2 mm were weighed. Change in weight before and after dropping was expressed as drop resistance [23]. A total of 50 pellets were placed in a rotating drum with an internal diameter and length of 80 and 120 mm, respectively. The drum was equipped with two opposite inner baffles (10 × 120 mm) arranged perpendicularly to the cylinder wall. The rotation speed was set to 50 r.p.m. After 10 min, the biochar pellets were removed from the drum and screened with a 2 mm sieve. The percentage of the original samples that remained unbroken provided the durability [24]. The compression strength of each pellet was measured with an Instron machine. A single biochar pellet was placed between two horizontal plates and compressed at a rate of 25 mm min^−1^. A data logger connected to the machine recorded the force (*F*) applied on the biochar pellet. The maximum force was measured as the value for the compression strength when the biochar pellet was crushed [23].

Functional groups were examined by Fourier transform infrared (FT-IR) analysis (Nicolet 6700, America). Sample discs were prepared by mixing the dried samples with KBr powder at ambient temperature at a biochar/KBr ratio of 1 : 200.

We sputter-coated the samples with Pt to increase their conductivity for subsequent analysis by scanning electron microscopy (SEM). A JSM-6490LV scanning electron microscope (JEOL, Japan) was used for the analysis.

## 3. Results and discussions

### 3.1. Mass and energy yields of the biochar pellets

Pyrolysis temperature is the most important factor affecting thermal stability. Table 1 shows the elemental compositions (C, H, O, N and S) of the samples, results of the proximate analysis and mass and energy yields of the biochar pellets.

The mass and energy yields of the biochar pellets followed a decreasing trend at increasing temperature. The mass and energy yield decreased from 35.46% to 28.65% and from 50.17% to 45.52%, respectively, which were similar to the results of previous study on powder samples [8,12]. By contrast, the HHVs of the biochar pellets increased from 21.86 to 24.55 MJ kg^−1^. The results demonstrated that biochar pellets can replace lignite and subbituminous coal, whose HHVs were 16.38–28.10 MJ kg^−1^ [25,26].

Increasing pyrolysis temperature considerably resulted in the increase in the C content, and the decrease in the H and O contents of the biochar pellets. The total C content of the raw pellet was 40.02%, whereas those of the biochar pellets were 56.46%, 57.14%, 58.85%, 59.29% and 60.84% at 400°C, 450°C, 500°C, 550°C and 600°C, respectively. The results indicated that biochar pellets became more carbonaceous as the temperature increased. The results are consistent with those of Chen & Lin [15]

**Figure 2.**
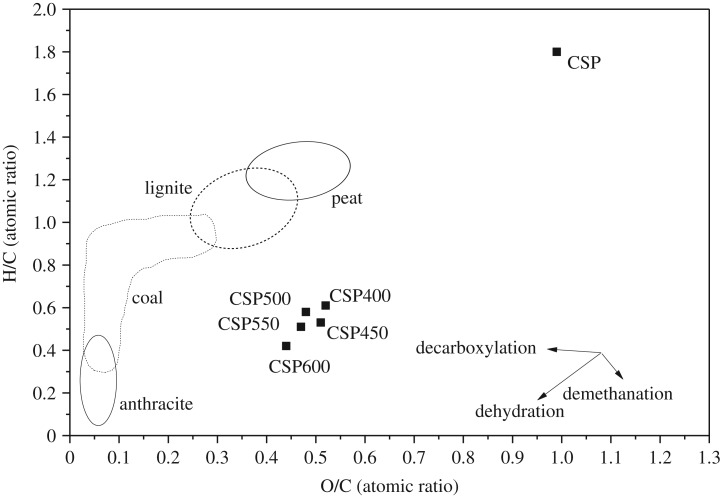
Van Krevelen diagram for raw pellet and biochar pellets at different temperatures.

The H/C and O/C atomic ratios of the corn straw pellets and their biochar pellets are shown in the Van Krevelen diagram (figure 2), which indicates that the evolution of the H/C and O/C atomic ratios from raw pellets to biochar pellets followed the paths of decarboxylation and dehydration reactions. At increased temperature, the H/C and O/C atomic ratios of the biochar were close to the origin, indicating that the biochar pellets have better fuel characteristics than raw biomass. For the purpose of estimating fuel properties, the H/C and O/C atomic ratios of the anthracite, coal, lignite and peat are shown in figure 2 [27].

### 3.2. Hydrophobicity

Water resistance of all pellets was used as an indicator for hydrophobicity. The high moisture content easily led to the fragmentation of the biomass pellets. This fragmentation can result in difficulties in the storage and transportation of biomass pellets. The results for the hydrophobicity of raw pellets and biochar pellets are shown in table 2. With regard to EMC before immersion in water, the hydrophobicity of biochar pellets was improved by pyrolysis, but decreased at pyrolysis temperatures from 400 to 600°C. Compared with the EMC of raw pellets, which was 6.85% when the RH was 42%, the EMC of the biochar pellets increased from 3.28 to 5.89%. When the RH was 75%, the EMCs of the raw pellets were all 8.53%, while those of the biochar pellets increased from 5.22 to 7.39%. To evaluate the strength of the pellets against the water resistance, we immersed the raw pellets and biochar pellets in water for 2 h. The raw pellets rapidly inflated and separated into fine particles within 10 min after immersion in water. At all pyrolysis temperatures, the biochar pellets sustained their shapes and structures and remained intact even after immersion in water for more than 2 h. The CSP600 floated on the surface of the water, and then submerged in water after 30 min. The highest EMC of the biochar pellets after immersion in water were observed at a pyrolysis temperature of 600°C. These results are opposite to those of some researchers [8,23], who observed that increase in pyrolysis temperature lowers the hydrophility of biochar. The reason may be that the pore structures of biochar pellets are damaged by the contraction of the biomass pellets during pyrolysis and the hydrophobicity of the biochar pellets is slightly increased at higher temperatures.

**Table 2.** The hydrophobicity of corn straw pellets and biochar pellets. Deviations are within 5%.

samples	the EMC before immersion in water (%)		the EMC after immersion in water (%)	BET surface area (m^2^/g)
	RH 42%	RH 75%		
CSP	6.85	8.53	—	
CSP400	3.28	5.22	26.11	4.26
CSP450	4.26	5.64	27.74	5.97
CSP500	4.95	6.12	27.80	12.61
CSP550	5.14	6.61	28.51	24.38
CSP600	5.89	7.39	29.42	29.60

The chemical compositions and structures of pellets have a strong influence on their hydrophobic behaviour. The pyrolysis process eliminates hemicelluloses, celluloses and lignins from biomass samples, which influenced the capacity for water sorption [23]. Hence, the EMCs of the biochar pellets before immersion in water were less than those of the raw pellets. The EMC of biochar pellets before and after immersion in water increased with increasing pyrolysis temperature. The reason is that the micropores of the biochar pellets increase with increasing pyrolysis temperature and absorb more free water [28] (table 2).

### 3.3. Mechanical resistance

The mechanical resistance is a good method for evaluating the strengths and qualities of biochar pellets during their transport and storage. Drop resistance and durability can be used in predicting the ability of biochar pellets to remain intact during transport and storage [23,24]. The compression strength of biochar pellets also indicates the internal bonding strength or the maximum force that a pellet can withstand before its rupture during storage. Drop resistance, durability and compression strength values of the raw and biochar pellets are shown in table 3.

**Table 3.** The mechanical resistance of raw pellets and biochar pellets. Deviations are within 5%.

samples	drop resistance (%)	durability (%)	compression strength (kN)
CSP	99.22	99.23	0.8318
CSP400	91.98	31.82	0.1694
CSP450	94.05	40.73	0.2034
CSP500	94.11	42.83	0.2202
CSP550	95.24	44.11	0.2345
CSP600	95.98	45.21	0.2459

The drop resistance and durability of biochar pellets increased with increasing pyrolysis temperature. However, the drop resistance and durability were found to be lower than those of the raw pellets. The durability ranged from 31.82 to 45.21%, which was far below the untreated sample's value (99.23%). Similarly, the compression strength of the biochar pellets increased with increasing pyrolysis temperature, but remained lower than those of raw pellets in most cases. A possible reason for the low durability and compression strength of the biochar pellets is the presence of pores, which might have reduced the mechanical properties of the pellets [29]. The average pore diameters decreased when pyrolysis temperature increased from 400 to 600°C [8,30] owing to the stable spatial structure. The results indicated that biochar pellets demonstrated excellent resistance, although the durability and compression strength were reduced to some extent.

### 3.4. Fourier transform infrared analysis

**Figure 3.**
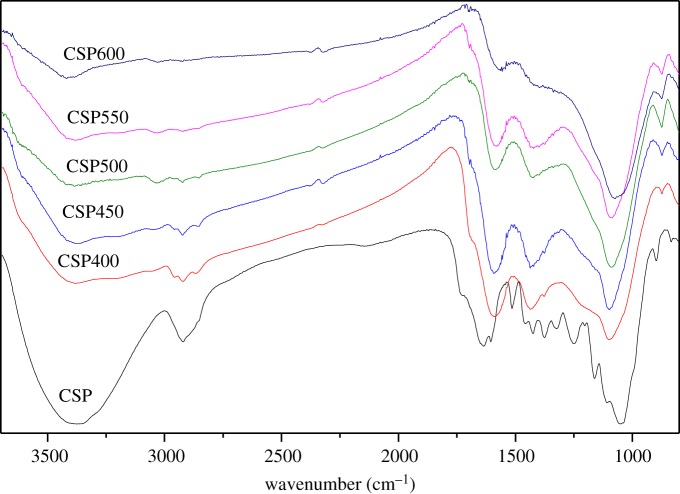
FT-IR spectra of corn straw and biochar pellets at different temperatures.

The FT-IR spectra are shown in figure 3 at various temperatures. The results of the FT-IR spectra for the raw corn straw had complex transmittance bands, and biochars obtained were similar to the raw sample. However, some functional groups weakened or disappeared. At increased temperature, the biochars became different in terms of the FT-IR spectra. At 600°C, functional groups in the char were nearly non-existent. A broad band at 3380 cm^−1^ was attributed to the stretching of the hydrogen-bonded −OH group. This peak weakened when the temperature was increased to 600°C. This condition may be attributed to the dehydration of the biomass. Meanwhile, a large amount of water was released. The bands attributable to aliphatic −CH_2_ and aromatic −CH_3_ (2921, 2855 cm^−1^) also decreased to zero as the temperature increased to 550 or 600°C. The decrease was possibly due to the breaking of the weak bonds between the C and H atoms of the alkyl groups. The breaking of the C–H functional groups promoted the release of CH_4_, C_2_H_6_ and C_2_H_4_ and resulted in the presence of CH_4_, C_2_H_6_ and C_2_H_4_ in gaseous products [31].

The bands at 1590 and 1436 cm^−1^ represented the stretching vibration of C=C and C=O in lignin, and the intensities of these bands decreased at 600°C, where few peaks were observed except aromatic C=C and C=O. The results indicated that the aromatization degree of biochar gradually increased. The bands at 1060 and 875 cm^−1^ were due to the stretching of C–O–C, and Si–O–Si groups, respectively. No obvious change in their peaks was observed because of their stable chemical bonds.

### 3.5. Scanning electron microscopy analysis

**Figure 4.**
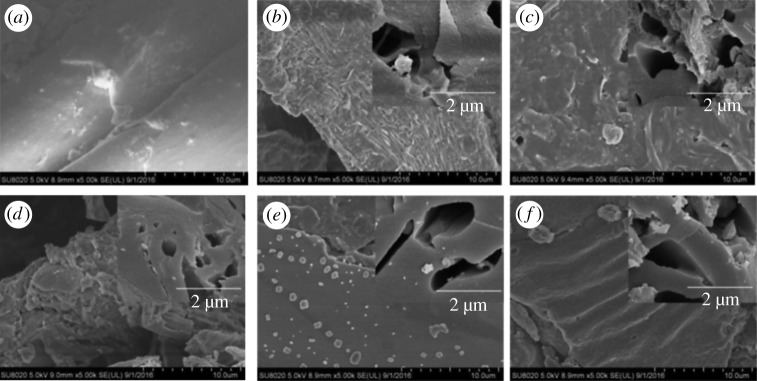
SEM image of raw pellet and biochar pellets at different temperatures: (*a*) CSP, (*b*) CSP400, (*c*) CSP450, (*d*) CSP500, (*e*) CSP550 and (*f*) CSP600.

The physical structures of biochar pellets at various temperatures are shown in figure 4. The biochar pellets displayed compact structures and regular shapes, although the structures varied at increasing temperatures. The structure was stacked together layer-by-layer because of compression during the pelletization process and remained as a multi-layered structure in the longitudinal direction. Meanwhile, the surface was quite clear.

Figure 4 clearly provides the biochar porous structure under SEM. The pore sizes of biochar pellets were heterogeneous, and the pore size of CSP400 was smaller. Highly porous biochar pellets have the benefit of adsorption sites for ions and provide space for nutrients and water retention [32].

## 4. Conclusion

Biochar pellets generated from the pyrolysis of corn straw pellets were studied under different temperatures (400, 450, 500, 550 and 600°C). The HHVs of the biochar pellets increased from 21.86 to 24.55 MJ kg^−1^ at increased temperature. Biochar pellets can reduce the costs and problems associated with transport and storage of biomass because of its stable quality, strong weatherability and suitability to long-term storage. The excellent HHVs and physico-chemical properties of the biochar pellets demonstrated that biochars can replace lignite and subbituminous coal. Meanwhile, some functional groups weakened or disappeared in the biochar pellets, which displayed compact structures.

## Data accessibility

This article does not contain any additional data.

## References

[1] Roy MM, Corscadden KW 2012 An experimental study of combustion and emissions of biomass briquettes in a domestic wood stove. Appl. Energy 99, 206–212. (10.1016/j.apenergy.2012.05.003)

[2] Anupam K, Sharma AK, Lal PS, Dutta S, Maity S 2016 Preparation, characterization and optimization for upgrading *Leucaena leucocephala* bark to biochar fuel with high energy yielding. Energy 106, 743–756. (10.1016/j.energy.2016.03.100)

[3] Apaydın-Varol E, Pütün AE 2012 Preparation and characterization of pyrolytic chars from different biomass samples. J. Anal. Appl. Pyrol. 98, 29–36. (10.1016/j.jaap.2012.07.001)

[4] Lee Y, Park J, Ryu C, Gang KS, Yang W, Park YK 2013 Comparison of biochar properties from biomass residues produced by slow pyrolysis at 500°C. Bioresour. Technol. 148, 196–201. (10.1016/j.biortech.2013.08.135) 24047681

[5] Li H, Jiang LB, Li CZ, Liang J, Yuan XZ, Xiao ZH, Xiao ZH, Wang H 2015 Co-pelletization of sewage sludge and biomass: the energy input and properties of pellets. Fuel Process. Technol. 132, 55–61. (10.1016/j.fuproc.2014.12.020)

[6] Garcia-Maraver A, Rodriguez ML, Serrano-Bernardo F, Diaz LF, Zamorano M 2015 Factors affecting the quality of pellets made from residual biomass of olive trees. Fuel Process. Technol. 129, 1–7. (10.1016/j.fuproc.2014.08.018)

[7] Roni MS, Eksioglu SD, Searcy E, Jacobson JJ 2014 Estimating the variable cost for high-volume and long-haul transportation of densified biomass and biofuel. Trans. Res. Part D Trans. Environ. 29, 40–55. (10.1016/j.trd.2014.04.003)

[8] Irfan M, Chen Q, Yan Y, Pang R, Lin Q, Zhao X 2016 Co-production of biochar, bio-oil and syngas from halophyte grass (*Achnatherum splendens*, L.) under three different pyrolysis temperatures. Bioresour. Technol. 211, 457–463. (10.1016/j.biortech.2016.03.077) 27035478

[9] Volpe R, Messineo A, Millan M, Volpe M, Kandiyoti R 2015 Assessment of olive wastes as energy source: pyrolysis, torrefaction and the key role of H loss in thermal breakdown. Energy 82, 119–127. (10.1016/j.energy.2015.01.011)

[10] Yang J, Chen H, Zhao W, Zhou J 2016 TG-FTIR-MS study of pyrolysis products evolving from peat. J. Anal. Appl. Pyrol. 117, 296–309. (10.1016/j.jaap.2015.11.002)

[11] Kim YM, Kim S, Han TU, Park YK 2014 Pyrolysis reaction characteristics of Korean pine (*Pinus Koraiensis*) nut shell. J. Anal. Appl. Pyrol. 110, 435–441. (10.1016/j.jaap.2014.10.013)

[12] Tag AT, Duman G, Ucar S, Yanik J 2016 Effects of feedstock type and pyrolysis temperature on potential applications of biochar. J. Anal. Appl. Pyrol. 120, 200–206. (10.1016/j.jaap.2016.05.006)

[13] Kung CC, Zhang N 2015 Renewable energy from pyrolysis using crops and agricultural residuals: an economic and environmental evaluation. Energy 90, 1532–1544. (10.1016/j.energy.2015.06.114)

[14] Nunes LJR, Matias JCO, Catalão JPS 2014 A review on torrefied biomass pellets as a sustainable alternative to coal in power generation. Renew. Sustain. Energy Rev. 40, 153–160. (10.1016/j.rser.2014.07.181)

[15] Chen WH, Lin BJ 2016 Characteristics of products from the pyrolysis of oil palm fiber and its pellets in nitrogen and carbon dioxide atmospheres. Energy 94, 569–578. (10.1016/j.energy.2015.11.027)

[16] Yan W, Chen ZH, Sheng KC 2015 Carbonization temperature and time improving quality of charcoal briquettes. Trans. CSAE 31, 245–249. (doi:10.11975/j.issn.1002-6819.2015.24.037)

[17] Zhou C, Zhang Q, Arnold L, Yang W, Blasiak W 2013 A study of the pyrolysis behaviors of pelletized recovered municipal solid waste fuels. Appl. Energy 107, 173–182. (10.1016/j.apenergy.2013.02.029)

[18] Basu P, Rao S, Acharya B, Dhungana A 2013 Effect of torrefaction on the density and volume changes of coarse biomass particles. Can. J. Chem. Eng. 91, 1040–1044. (10.1002/cjce.21817)

[19] Paulauskas R, Džiugys A, Striūgas N 2015 Experimental investigation of wood pellet swelling and shrinking during pyrolysis. Fuel 142, 145–151. (10.1016/j.fuel.2014.11.023)

[20] Ghiasi B, Kumar L, Furubayashi T, Lim CJ, Bi X, Chang SK 2014 Densified biocoal from woodchips: is it better to do torrefaction before or after densification? Appl. Energy 134, 133–142. (10.1016/j.apenergy.2014.07.076)

[21] Chen M, Zhao J, Xia L 2008 Enzymatic hydrolysis of maize straw polysaccharides for the production of reducing sugars. Carbohydr. Polym. 71, 411–415. (10.1016/j.carbpol.2007.06.011)

[22] Jin W, Xu X, Yang G, Yang F, Gang W 2014 Anaerobic fermentation of biogas liquid pretreated maize straw by rumen microorganisms *in vitro*. Bioresour. Technol. 153, 8–14. (10.1016/j.biortech.2013.10.003) 24326083

[23] Kambo HS, Dutta A 2014 Strength, storage, and combustion characteristics of densified lignocellulosic biomass produced via torrefaction and hydrothermal carbonization. Appl. Energy 135, 182–191. (10.1016/j.apenergy.2014.08.094)

[24] Gil MV, Oulego P, Casal MD, Pevida C, Pis JJ, Rubiera F 2010 Mechanical durability and combustion characteristics of pellets from biomass blends. Bioresour. Technol. 101, 8859–8867. (10.1016/j.biortech.2010.06.062) 20605093

[25] Frau C, Ferrara F, Orsini A, Pettinau A 2015 Characterization of several kinds of coal and biomass for pyrolysis and gasification. Fuel 152, 138–145. (10.1016/j.fuel.2014.09.054)

[26] Hao L, Xia S, Ma P 2016 Thermogravimetric investigation of the co-combustion between the pyrolysis oil distillation residue and lignite. Bioresour. Technol. 218, 615–622. (10.1016/j.biortech.2016.06.104) 27416511

[27] Reza MT, Wirth B, Lüder U, Werner M 2014 Behavior of selected hydrolyzed and dehydrated products during hydrothermal carbonization of biomass. Bioresour. Technol. 169, 352–361. (10.1016/j.biortech.2014.07.010) 25063978

[28] Zornoza R, Morenobarriga F, Acosta JA, Muñoz MA, Faz A 2015 Stability, nutrient availability and hydrophobicity of biochars derived from manure, crop residues, and municipal solid waste for their use as soil amendments. Chemosphere 144, 122–130. (10.1016/j.chemosphere.2015.08.046) 26347934

[29] Li H, Liu X, Legros R, Bi XT, Lim CJ, Sokhansanj S 2012 Pelletization of torrefied sawdust and properties of torrefied pellets. Appl. Energy 93, 680–685. (10.1016/j.apenergy.2012.01.002)

[30] Chen Y, Yang H, Wang X, Zhang S, Chen H 2012 Biomass-based pyrolytic polygeneration system on cotton stalk pyrolysis: influence of temperature. Bioresour. Technol. 107, 411–418. (10.1016/j.biortech.2011.10.074) 22209443

[31] Zhao Y, Feng D, Zhang Y, Huang Y, Sun S 2015 Effect of pyrolysis temperature on char structure and chemical speciation of alkali and alkaline earth metallic species in biochar. Fuel Process. Technol. 141, 54–60. (10.1016/j.fuproc.2015.06.029)

[32] Rout T, Pradhan D, Singh RK, Kumari N 2016 Exhaustive study of products obtained from coconut shell pyrolysis. J. Environ. Chem. Eng. 4, 3696–3705. (10.1016/j.jece.2016.02.024)

